# A War Emergency Benevolent Fund

**Published:** 1917-07-14

**Authors:** 


### A WAR EMERGENCY BENEVOLENT FUND.

Equality of sacrifice for patriotic ends is a stimulating ideal, and it lends itself without discussion to the claims of natural justice. But, as is the case with many ideals, its translation into practice falls far short of the perfect scheme. Yet to profess a lower aim would be both ignoble and a hindrance to accomplishment. The high endeavour may not reach exactly the goal, but without it there would be a more conspicuous failure. These doctrines find apt illustration in the position occupied by the members of the medical profession relative to the claims of national service created by the war. While some have made large sacrifices and run great risks, others have been able to continue a smooth career. Differences of age, of health, and of circumstance have separated the groups and have exercised their inexorable sway. When the money test is applied neither the eldest nor the youngest has fared so badly. The one has pursued his usual work, merely pressed, as his fellow-citizens, by added taxes and increased expenses; the other has passed from his school to a relatively large income, qualified by a greater or less risk from shot and shell. But between these two extremes there are many practitioners who have suffered, and will yet suffer, a heavy monetary loss. Settled in practice for a moderate number of years, with hostages given to fortune in the shape of wives and children, and with permanent charges of rent, insurance, and taxes which cannot be relaxed, these men have been carried away from their daily work by the military machine, and they receive for their services payment far from sufficient to meet the expenses of their domestic responsibilities. But this is not all. Upon leaving the Army at the end of the war their military pay, of course, will cease, and only too many of them will return to find that their practices have almost or completely disappeared. There will be lean years before their former positions can be regained. Is there not a plain demand that some effort should be made to meet this situation and that by the intervention of those who have felt no financial penalty an attempt should be made to distribute the burden of sacrifice?

Such is the aim of the War Emergency Fund established by the Royal Benevolent Fund. Here the authorities have acted and have not been content with pious aspirations, and in these columns we welcome their efforts as being in accordance with the judgment we expressed in the early days of the war. Not less than £25,000 is needed to secure even partial help. It is an obligation of honour that this claim be met. The profession must tax itself for the purpose, lest colleagues and the wives and children of colleagues suffer beyond measure for an answer to the clear call of duty. There may be members of the public who as an expression of gratitude will gladly contribute to such a Fund. But the first message is to the profession, and an answer in greater or less amount is due from every member who has held, in spite of the war, a substantially undisturbed financial course. Of the administration of such a Fund by the Royal Medical Benevolent Fund it is not necessary to speak. Efficiency, economy, good faith, and knowledge are in the guarantee. What is needed is practical sympathy and co-operation in the shape of donations, and these should be sent to the Honorary Secretary, War Emergency Fund, 11 Chandos Street, Cavendish Square, W. 1. We hope to see many attempts to realise in action the equality of sacrifice on the excellence of which all are agreed.

